# Causal perception is central in electromagnetic hypersensitivity - a commentary on “Electromagnetic hypersensitivity: a critical review of explanatory hypotheses’’

**DOI:** 10.1186/s12940-020-00652-4

**Published:** 2020-11-25

**Authors:** Christoph Boehmert, Michael Witthöft, Omer Van den Bergh

**Affiliations:** 1 Federal Office for Radiation Protection, Oberschleißheim, Germany; 2 Department for Clinical Psychology, Psychotherapy, and Experimental Psychopathology, Johannes Gutenberg University Mainz, Mainz, Germany; 3 Health Psychology, University of Leuven, Leuven, Belgium

**Keywords:** Electromagnetic hypersensitivity, IEI-EMF, Nocebo, Attribution, Predictive processing

## Abstract

We highly welcome and appreciate the paper of Dieudonné, 2020 (10.1186/s12940-020-00602-0) on the important but frequently neglected topic of hypersensitivity towards electromagnetic fields (EHS). We agree with the author that the electromagnetic hypothesis (that EHS is caused by exposure to electromagnetic fields) appears scientifically largely unfounded and that other theoretical approaches focussing on psychological processes are more plausible and promising. In the view of the author, two such approaches exist, namely a “cognitive hypothesis” (derived from the comprehensive model by Van den Bergh et al., 2017) and an “attributive hypothesis” as suggested by the author. In this commentary, we want to argue (a) that the distinction between the cognitive and the attributive hypothesis is inaccurate at the conceptual level; (b) that the distinction is also misleading at the mechanistic level, due to an incorrect interpretation of the evidence related to the cognitive hypothesis; and (c) that, by using the term “cognitive hypothesis”, the existing comprehensive model is inappropriately narrowed down without fully appreciating its explanatory power for the phenomena subsumed under both the cognitive and attributive hypothesis. Therefore, the original term “comprehensive model” should be used rather than the label “cognitive hypothesis”.

We highly welcome and appreciate this contribution on the important but frequently neglected topic of hypersensitivity towards electromagnetic fields (EHS). Scientific debate about the most appropriate theoretical framework for EHS can only help to better understand this puzzling condition and to translate this knowledge into innovative and effective treatments.

We agree with the author that the electromagnetic hypothesis (that EHS is caused by exposure to electromagnetic fields, EMF) appears scientifically largely unfounded and that other theoretical approaches focussing on psychological processes are more plausible and promising. In the view of the author, two such approaches exist, namely a “cognitive hypothesis” (CH, derived from the comprehensive model, [1]) and an “attributive hypothesis” (AH, [2]). In this commentary, we want to argue (a) that the distinction between the cognitive and the attributive hypothesis is inaccurate at the conceptual level; (b) that the distinction is also misleading at the mechanistic level, due to an incorrect interpretation of the evidence related to the CH; and (c) that, by using the term CH, the comprehensive model is inappropriately narrowed down without fully appreciating its explanatory power for the phenomena subsumed under both the cognitive and attributive hypothesis. Therefore, the original term “comprehensive model” (CM) should be used rather than the label “cognitive hypothesis” (CH).

Ad (a): According to the author, the CH explains symptoms of EHS through a nocebo mechanism (opposite of placebo, resulting from expectations of harm), whereas the AH explains them as retrospective causal explanations (attributions) for existing medically unexplained symptoms. We believe it is misleading to consider these two hypotheses as distinct. This is because the core of both nocebo and attribution is a cognitive process representing a belief about a cause-effect relationship. Once this causal belief about EMF and bodily harm is established, it will act as a cognitive frame, both to expect the occurrence of symptoms in response to perceived EMF exposure and to facilitate attributing (unexplained) symptoms to EMF. This means that attributions, once consolidated in a belief about a cause-effect relationship, will also generate expectations about symptoms on subsequent exposures to EMF and thereby, possibly, nocebo effects. It is therefore conceptually inaccurate to introduce a distinction between CH and AH. Moreover, causal beliefs and (mis)attribution represent an essential part *within* the CM (see Fig. 1 [3];).

**Fig. 1:**
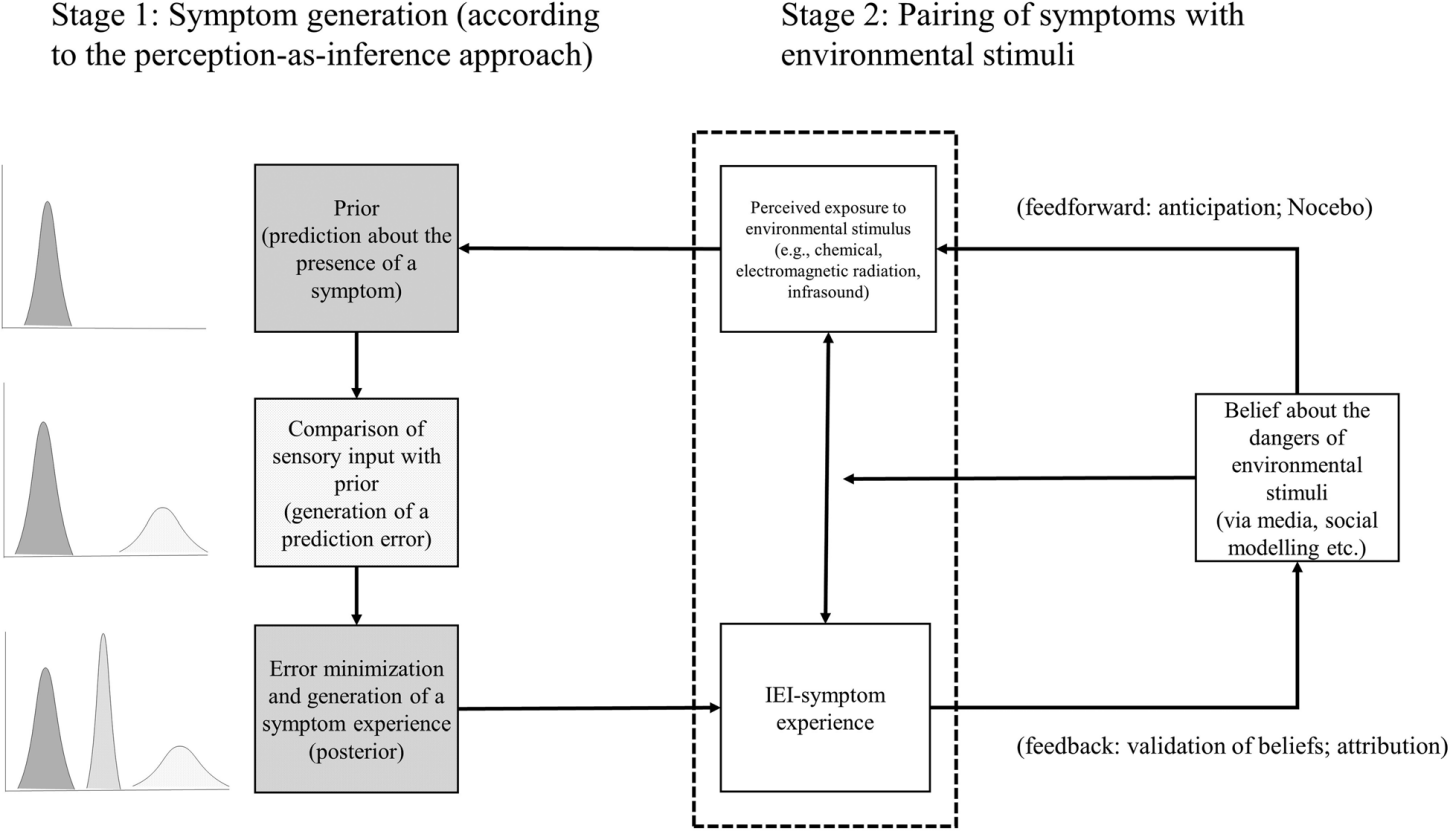
A simplified illustration of a perception-as-inference approach to idiopathic environmental intolerance (IEI). Symptoms of IEI are hypothesized to result from somatic symptom experiences (stage 1) that become associated with environmental stimuli (stage 2). Once symptom-stimuli associations have been formed (e.g., via classical conditioning, social modelling), the perception of environmental stimuli is able to foster the formation of strong and precise priors that are able to determine conscious symptom perceptions in the posterior model. IEI-symptom experiences reinforce IEI related beliefs in memory (feedback route) and shape the priors of the symptom-perception model for upcoming symptom perception episodes (feedforward route)

Ad (b): Mechanistically, an important issue is how beliefs about cause-effect relationships come about. According to the CM, these relationships may emerge from a variety of sources including (Pavlovian) conditioning, verbal information and social modelling, which may differentially impact the level of awareness (i.e. some expectations and beliefs may be hard to explicitly verbalize). The CM also describes a dynamic evolution of EHS which may eventually change (a) critical processes and (b) the order of events. For example, in some cases stress-related physiological reactivity (e.g. from hyperventilation) may prime a person to search in the environment for salient potential causes of the felt bodily sensations (for instance, a novel WLAN-router) and/or raise sensitivity for sources of EMF after media coverage and/or activist information. In other cases, media and/or social influences may first prime sensitivity for EMF sources by increasing the awareness of abundantly present EMF sources, making it more likely to perceive causal links of EMF with occasional stress-related physiological reactivity. However, once a stable causal belief has been established, it may act as a nocebo inducing symptoms without necessarily requiring physiologic input, as is documented in experiments [1].

In view of the examples above, several misunderstandings of the CM by the author become obvious. First, it is claimed that experimental studies demonstrating symptoms after expectation induction are not convincing, because the effects have not been shown to be long lasting. It should be noted that laboratory experiments are intended to demonstrate a mechanistic principle, not to create a patient. So, experiments will never completely mimic clinical processes. Nevertheless, mild nocebo effects from WLAN have recently been shown to last at least a week [4]. In addition, contrary to the author’s claim, moderators of nocebo effects related to negative affect, and clinical expressions such as anxiety and depression, have been documented in the area of pain [5] and multiple chemical sensitivity [1]. Second, it is inaccurate to suggest that the CH critically relies on media coverage of EMF. Media reports are only one example of how cause-effect relationships can be primed as frames to construct reality. As demonstrated for nocebo/placebo, other equally important and sometimes more powerful sources of information can be personal experience of temporal contingencies between symptom episodes and perception of EMF sources, communication with relatives, physicians, etc., or also the observation of behaviours [6]. Third, it is inaccurate to suggest that, according to the CM, symptoms always start with a belief of EMF being present. The above description shows that different orders of events are consistent with the CM to induce the perception of a cause-effect relationship, and that from then on nocebo processes may cause symptoms without afferent physiologic input. In other words, the limitations of the CH as explained by the author in his paper do not apply.

There are several other misunderstandings, such as “*EHS is thus explained [by the CM] analogically to hypochondria, classically attributed to a phenomenon of somatosensory amplification [..], by a model where misplaced fears are no longer related to the body but to the environment.”* The above comparison of EHS with hypochondria is inaccurate because EHS does not represent an anxiety disorder and fear is not the crucial process. Also, the CM explicitly differs from the somatosensory amplification account, because (1) the latter necessarily requires afferent somatic input as a prerequisite for symptom experiences whereas the CM does not, and (2) the CM suggests that selective attention as well as (negative) interpretive processes are not necessarily causes, but most often consequences of the symptom generation process itself.

Ad (c): The CM is inappropriately narrowed down to a “cognitive hypothesis”. The CM relies on a novel neurobiological conceptualization – predictive processing – about how the brain constructs an adaptive model of the (internal and external) world using the spatial and temporal patterning of its own neural activity. Such a model of the world is constructed across multiple hierarchical layers of the brain, where incoming information interacts with predictions that are automatically generated by the brain. The resulting prediction errors are subsequently propagated through the brain in an error minimizing process. The assumption is that conscious awareness emerges for the model that is associated with the least overall level of prediction error. An important implication is that reality as it is experienced is a construction that, depending on reliability parameters, can be either closer to the generated predictions or to the incoming information.

Applied to how bodily symptoms emerge in conscious awareness, the CM is a specific exemplar of a novel generic model of symptom perception with a wide explanatory scope (see Fig. 1 [3];). First, it is able to explain when and why there is a close correspondence between the experience of symptoms and objective physiology and when they are likely to diverge. For example, it is well documented that symptoms in chronic conditions are often only loosely coupled with objective disease indicators. Second, it explains also when and why symptom reports can be completely uncoupled from physiological dysfunction. Dieudonné rightly draws attention to a large proportion of symptom reports that remain medically unexplained in both primary and secondary care and in his AH he describes the attribution of these symptoms to a cause as an attempt to make sense of the symptoms. However, the CM also explains how medically unexplained symptoms come about, in addition to describing how they are compellingly linked to environmental factors in a process of meaningful perception. Third, while the AH only explains EHS, the CM explains EHS as well as other conditions that are characterized by a scientifically unfounded causal link between symptoms and an environmental factor, such as multiple chemical sensitivities, infrasound hypersensitivity and various unfounded food and other allergies.

In sum, we believe that presenting the AH as a separate hypothesis to understand EHS creates confusion and ignores existing theoretical and empirical work.

## Data Availability

No original data are presented.

## Abbreviations

AH: Attributive Hypothesis
CH: Cognitive Hypothesis
CM: Comprehensive Model
EHS: Hypersensitivity towards Electromagnetic Fields
EMF: Electromagnetic Field(s)
IEI: Idiopathic Environmental Intolerance
WLAN: Wireless Local Area Network
